# Exploration of Analgesia with Tramadol in the Coxsackievirus B3 Myocarditis Mouse Model

**DOI:** 10.3390/v13071222

**Published:** 2021-06-24

**Authors:** Sandra Pinkert, Meike Kespohl, Nicolas Kelm, Ziya Kaya, Arnd Heuser, Karin Klingel, Antje Beling

**Affiliations:** 1 Institute of Biochemistry, Charité–Universitätsmedizin Berlin, Corporate Member of Freie Universität Berlin and Humboldt-Universität zu Berlin, 10117 Berlin, Germany; sandra.pinkert@charite.de (S.P.); meike.kespohl@charite.de (M.K.); nicolas.kelm@charite.de (N.K.); 2 Deutsches Zentrum für Herz-Kreislauf-Forschung (DZHK), Partner Site Berlin, 10115 Berlin, Germany; 3 Berlin Institute of Health at Charité (BIH), 10117 Berlin, Germany; 4 Kardiologie, Angiologie und Pneumologie, Medizinische Klinik für Innere Medizin III, Universitätsklinikum Heidelberg, 69120 Heidelberg, Germany; ziya.kaya@med.uni-heidelberg.de; 5 Deutsches Zentrum für Herz-Kreislauf-Forschung (DZHK), Partner Site Heidelberg, 69120 Heidelberg, Germany; 6 Animal Phenotyping Platform, Max-Delbrueck-Center for Molecular Medicine, 13125 Berlin, Germany; heuser@mdc-berlin.de; 7 Cardiopathology, Institute for Pathology and Neuropathology, University Hospital Tuebingen, 72076 Tuebingen, Germany; karin.klingel@med.uni-tuebingen.de

**Keywords:** infection, myocarditis, pancreatitis, refinement, analgesia

## Abstract

Infection of mice with Coxsackievirus B3 (CVB3) triggers inflammation of the heart and this mouse model is commonly used to investigate underlying mechanisms and therapeutic aspects for viral myocarditis. Virus-triggered cytotoxicity and the activity of infiltrating immune cells contribute to cardiac tissue injury. In addition to cardiac manifestation, CVB3 causes cell death and inflammation in the pancreas. The resulting pancreatitis represents a severe burden and under such experimental conditions, analgesics may be supportive to improve the animals’ well-being. Notably, several known mechanisms exist by which analgesics can interfere with the immune system and thereby compromise the feasibility of the model. We set up a study aiming to improve animal welfare while ensuring model integrity and investigated how tramadol, an opioid, affects virus-induced pathogenicity and immune response in the heart. Tramadol was administered seven days prior to a CVB3 infection in C57BL/6 mice and treatment was continued until the day of analysis. Tramadol had no effect on the virus titer or viral pathogenicity in the heart tissue and the inflammatory response, a hallmark of myocardial injury, was maintained. Our results show that tramadol exerts no disruptive effects on the CVB3 myocarditis mouse model and, therefore, the demonstrated protocol should be considered as a general analgesic strategy for CVB3 infection.

## 1. Introduction

Myocarditis is an inflammatory disease of the heart, frequently induced by virus infections [1]. In order to investigate pathogenic mechanisms of virus-induced myocarditis and subsequent inflammatory processes with alterations in the extracellular matrix of the heart, most researchers rely on the Coxsackievirus B3 (CVB3) myocarditis model in mice [2,3,4,5]. This model mimics the general aspects of viral myocarditis in humans and shows primary virus replication in the intestinal organs (enterovirus [6] and adenovirus [7]), the lung (influenza virus, some indication also for SARS-CoV2 [8,9,10]) or skin (rubella virus [11]), with viruses spreading via the blood stream (viremia) and subsequent infections of the heart. It was established about 60 years ago by intraperitoneal (i.p.) infection of mice with CVB3 [12]. Induced by viral infection of the cardiomyocytes, the pathogenesis of viral myocarditis is driven by virus-caused cell destruction and the infection-triggered activation of the immune system, whereby inflammatory processes are of particular importance for this model [2]. The sensing of viral replication intermediates by their cognate receptors triggers pro-inflammatory molecules and stimulates the infiltration of immune cells. Natural killer (NK) cells and monocytes/macrophages represent classic infiltrating cells and, to a minor extent, B-lymphocytes and T-lymphocytes also invade. Undisputedly, the immune response is required for virus control, but there is also consensus that inflammatory processes drive cardiac damage and fibrotic remodeling [4,13].

In mice, the pancreas is the most vulnerable organ for CVB3 infection and mice develop pancreatitis in the first couple of days after infection [14]. Symptoms that can be at least partially attributed to pancreatitis are weight loss and stooped posture, with the latter resulting most likely from abdominal pain. In an earlier attempt at reducing or eliminating this health burden of the myocarditis model, we engineered a CVB3 strain that was attenuated for the infection of the pancreas and, in so doing, we achieved reduced animal suffering [15]. However, the pancreas-attenuated virus was less effective in provoking myocarditis and this constrained the experimental approach.

Here, we propose an alternative strategy for improving the well-being of experimental animals, which involves treating mice that were infected with the wild-type virus with an analgesic drug. In accordance with the animal welfare act of the European Union (Directive 2010/63/EU), the application of analgesia for infection and other inflammatory disease models may be considered as a suitable intervention to increase the well-being of the animals. Nevertheless, analgesics, such as nonsteroidal anti-inflammatory drugs, and also opioids exert immune modulating properties and these might affect the model’s defining characteristics. Among the many drugs available for the relief of pain, we focused our research on tramadol, which is an opioid that shows multifaceted immunomodulatory properties [16,17]. Tramadol is a centrally acting analgesic drug that is used for the treatment of intermediate to severe pain [18,19]. Its analgesic efficacy is based on a complex interaction between the adrenergic, the opiate, and the serotonin receptor systems [20]. Tramadol is a weak opioid agonist, with specific selectivity for the µ receptor, and a weak inhibitor of the re-uptake of norepinephrine and serotonin [21,22]. Tramadol shows no class-specific side-effects, such as respiratory depression, constipation, or sedation [20]. Moreover, the immunomodulatory properties of this opioid can be altered by introducing a preconditioning phase prior to the induction of pain. In contrast to biologically relevant immunomodulation, it was noted after a short-term application of this drug in mice studies that a prolonged treatment protocol showed no relevant immune modification, while the analgesic effect remained [22]. As a further advantage, tramadol can be administered orally via drinking water and further interventions, such as subcutaneous (s.c.) or intraperitoneal (i.p.) injections, required for other opioids and the associated additional health burdens to the existing infection are not necessary [23]. Here, we set up a study using an established analgesic dosage of 0.1 mg/mL tramadol in the mouse model of CVB3 myocarditis to investigate the impact of this analgesic strategy on the two main determinants of the myocarditis model, which are the the virus-triggered cytotoxicity and the inflammatory response.

## 2. Materials and Methods

### 2.1. Animals and Virus

Male C57BL/6J mice were purchased from Charles River, Germany and housed in the animal facility at the Charité University Medical Center. The study was performed in accordance with the German Animal Welfare Act, which is based on the Directive of the European Parliament and was approved by the Committee on the Ethics of Animal Experiments of Berlin state authorities—“Landesamt für Gesundheit und Soziales” (LaGeSo; permit number G0026/20). Myocarditis was induced in male mice by a intraperitoneal injection of 1 × 10^5^ plaque forming units (pfu) of a cardiotropic CVB3 variant (Nancy strain) [2]. For analgesia, Tramal^®^ (tramadol hydrochloride solution 100 mg/mL; Grünenthal GmbH, Aachen, Germany) 0.1 mg/mL was administered via drinking water (tap water) and was renewed daily. Tramadol treatment begun seven days before infection and was continued until the day of analysis. The untreated control group also had their water renewed daily. Water consumption rate with or without tramadol was estimated for each cage by relative daily weight reduction in the drinking bottles. Soaked food and water gel (2% SAFE^®^ hydro; J. Rettenmaier & Söhne GmbH + Co.KG, Rosenberg, Germany) were added to the cage beginning on the day of infection. In order to monitor the body weight during infection, animals were weighed daily beginning on the day of infection. 

A model-specific physical condition score (PCS) was used to monitor animal welfare. The PCS includes activity, facial expression, grooming, and appearance. Two blinded observers, using a score from one to five, evaluated each animal independently according to the following. Activity after opening the cage: (1) remained in the nest and inactive after removal to (5) highly active and climbing on the walls and lid of the cage. Facial expression/grimace scale: (1) nose bulge, squeezed eyes, and ears drawn back to (5) clear, open eyes, ears upright, and straight nose. Grooming: (1) shaggy and scruffy coat with dirty rectum to (5) clean and shiny coat. Body posture (1) strongly bent and hunched during walking to (5) normal stretched out body posture. The CVB3 infection model was analyzed on days 1.5, 3, 5 and 8 after infection. On the day of analysis, after echocardiography, blood was collected from isoflurane-anaesthetized mice and centrifuged at 10,000× *g* for 10 min at room temperature. Separated serum was then collected and stored at −80 °C. Organs were collected for further analysis, stored in HistoFix (1xPBS, 4% ROTI^®^Histofix; Carl Roth GmbH, Karlsruhe, Germany) at 4 °C (for flow cytometry), or immediately frozen in liquid nitrogen and stored at −80 °C.

### 2.2. Echocardiography

For echocardiography, mice were anesthetized with 1.5–2.0% isoflurane and kept warm on a heated platform during the procedure. Temperature and ECG were monitored continuously and cardiac function were assessed with a VisualSonic Vevo3100 High-Frequency Imaging System using a high-resolution (44 MHz) transducer. An experienced technician, who was not aware of the specific treatment of each mouse, performed all measurements. Standard imaging planes and measurements were obtained using standard M-mode. Functional and dimensional calculations are based on the parasternal long axis view of the left ventricle.

### 2.3. Quantification of Infectious CVB3 

The amounts of infectious virus in different tissues were determined by standard plaque assay on HeLa cells. Hela cells (ATCC CCL-2) were cultured in MEM (Life Technologies, Waltham, MA, USA) supplemented with 5% fetal calf serum (FCS) and 1% penicillin/streptomycin (Life Technologies). Plaque assays were performed in duplicates on 24-well cell culture plates, using 10-fold dilutions of homogenized organs to infect cell monolayers for 30 min at 37 °C. After incubation, supernatants were carefully discarded and cells were overlaid with Eagle’s-agar (MEM, 1% penicillin/streptomycin, 2.3 g/L NaHCO_3_, 9% fetal bovine serum (FBS), and 0.7% Difco Agar Noble (BD Bioscience, Heidelberg, Germany). Two to three days after infection, cells were stained with MTT (3-/4,5-dimethylthiazol-2-yl)-2,5-diphenyl tetrazolium bromide), incubated between two and four hours, and plaques were subsequently counted.

### 2.4. Histology

Tissue samples were fixed overnight, rinsed with PBS, and embedded in paraffin. In order to visualize immune cell infiltration and tissue necrosis, cross sections of 3–5 µm thickness were cut and stained with hematoxylin and eosin (HE). Evaluation was carried out by a pathologist as described elsewhere [24].

### 2.5. Isolation of Immune Cells from Mouse Tissues

Immune cells were obtained from the bone marrow, spleen, and heart. For the analysis of bone marrow cells, flushed bone marrow of one tibia and two femora for each mouse was passed through a 70 µm cell strainer and centrifuged (10 min, 310× *g*). For red blood cell lysis, cell pellet was re-suspended in 0.83% ammonium chloride (NH_4_Cl) in PBS and incubated for 3 min at room temperature. Cells were recovered by centrifugation, re-suspended in FACS buffer (1xPBS, 2% FBS, 2mM EDTA), and stored on ice until flow cytometry was conducted. Splenocytes were prepared by passing the spleen tissue through a 100 µm cell strainer, followed by RBC lysis as described above. In order to isolate immune cells from heart tissue, the heart was flushed with 15 mL PBS and a part of the ventricle (~20 mg) was stored in a wash medium (RPMI 1640, 2% FCS, 1% penicillin/streptomycin, 30 mM HEPES) at 4 °C until further preparation. Heart sections were minced and cells were extracted by digestion using 1 mg/mL collagenase type 2 (Worthington Biochemical Corporation, Lakewood, NJ, USA) and 0.15 mg/mL DNase I (Sigma-Aldrich, St. Louis, MO, USA) in a wash medium for 30 min at 37 °C under continuous agitation. EDTA was added at a concentration of 10 mM before samples were passed through a 70 µm cell strainer. Erythrocyte lysis was performed as described.

### 2.6. Flow Cytometry

Panel for cardiac flow cytometry staining: Cells corresponding to 20 mg heart tissue were incubated with Fc blocking reagent (Miltenyi, Bergisch Gladbach, Germany) at a concentration of 1:50 for 20 min at 4 °C. Cells were then stained with the antibodies listed below for 20 min at 4 °C while protected from light. Samples were washed with a FACS buffer (PBS with 2% FCS and 2 mM EDTA) and PBS (centrifugation: 3 min at 300× *g*) before Fixable Viability Dye eFluor 780 (eBioscience, San Diego, CA, USA) was applied according to the manufacturer’s instructions. Samples were fixed in 2% formaldehyde/PBS for 30 min at room temperature and washed with PBS prior to the addition of 123count eBeads (Thermo Fischer Scientific, Waltham, MA, USA) for the quantification of total cell number. Antibodies were purchased from BD (CD8 clone 53-6.7, MHCII clone I-A[B]AF6-120.1, CD3 clone 145-2C11) and Biolegend (San Diego, CA, USA) (F4/80 clone BM8, CD11b clone M1/70, Ly6G clone 1A8, CD11c clone N418, Ly6C clone HK1.4, B220 clone BD-R43-6B2, CD4 clone RM4-5, and CD45.2 clone 104). 

Panel for flow cytometry staining of spleen and bone marrow: Equal numbers of cells were stained as described above using antibodies from BD (CD137 clone AH2, NK1.1 clone PK136, CD4 clone RM4-5, CD8 clone 53-6.7, CD3 clone 145-2C11, CD86 clone GL1, and MHCII clone I-A[B]AF6-120.1) and Biolegend (B220 clone RA3-6B2, CD19 clone 6D5, CD69 clone H1.2F3, CD62L clone MEL-14, CD44 clone IM7, CD45.2 clone 104, F4/80 clone BM8, CD11b clone M1/70, Ly6G clone 1A8, CD11c clone N418, Ly6C clone HK1.4, B220 clone R43-6B2, CD206 clone CO68C2, CD169 clone 3D6.112, CD3 clone 145-2C11, CD49b clone DX5, Ter119 clone Ter119, and CCR7 clone 4B12). The subsequent staining procedure was dconducted as described above. Flow-cytometric analysis was conducted on a BD FACSymphony flow cytometer and data were analyzed using FlowJo V10.6.2 software (Ashland, Wilmington, DE, USA) by applying the gating strategy depicted in the Supplemental Figures S1–S3.

### 2.7. Quantitative Real-Time PCR

RNA was isolated using TRIzol (Ambion, Thermo Fischer Scientific) according to the manufacturer’s instructions. DNA contamination was removed by digestion with deoxyribonuclease I (Invitrogen, Thermo Fischer Scientific) at 37 °C for 15 min followed by a deactivation step at 65 °C for 10 min. RNA was reverse-transcribed by MLV Reverse Transcriptase (Promega, Madison, WI, USA) in combination with random hexamer primers (Roche, Basel, Switzerland). TaqMan polymerase chain reaction (PCR) was performed using primers and probes of TaqMan gene expression assays (Life Technologies, Thermo Fischer Scientific) as well as the following combinations of primers and probes: murine HPRT; forward primer 5′-ATC ATT ATG CCG AGG ATT TGG AA-3′, reverse primer 5′-TTG AGC ACA CAG AGG GCC A-3′, probe 5′-FAM-TGG ACA GGA CTG AAA GAC TTG CTC GAG ATG-3′ TAMRA, and CVB3; forward primer 5′-CCC TGA ATG CGG CTA ATC C-3′, reverse primer 5′-ATT GTC ACC ATA AGC AGC CA-3′, and probe 5′-FAM-TGC AGC GGA ACC G-MGB-3′. A StepOnePlus real-time PCR system was used for quantitative PCR (qPCR). HPRT served as an endogenous control and was used to calculate relative expression using the ΔCT method.

### 2.8. Quantification of Cardiac Troponin T 

Blood samples were collected, centrifuged and serum aliquots were stored at −80 °C until analysis. For cardiac Troponin T quantification, a Cobas E411 automated analyzer was used (Roche Diagnostics, Mannheim, Germany).

### 2.9. Statistics

Statistical analysis of the data was performed using GraphPad Prism v7.00 for Windows (GraphPad Software, La Jolla, CA, USA). If not indicated otherwise, data summaries are given as means ± SEM. Unpaired *t* tests were used for two-group comparisons after the outliers were identified using the ROUT method (Q = 1%). For multiple group comparison, two-way ANOVA was performed after outliers were identified using the ROUT method (Q = 1%). As post-hoc tests, Tukey’s multiple comparison was used to analyze virus effects over time, while Sidak’s multiple comparison was used to analyze differences between the two treatment groups. The significance threshold for all tests was set at the 0.05 level.

## 3. Results

**Figure 1:**
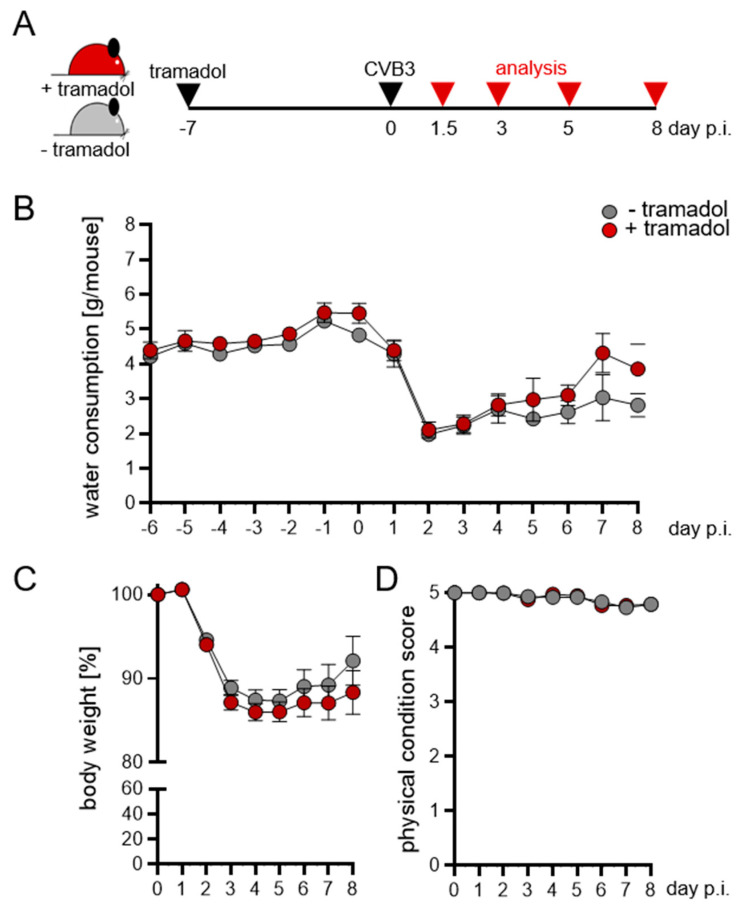
Impact of tramadol on water consumption and body weight. (**A**) Time course of the Coxsackievirus B3 (CVB3)-induced acute myocarditis model as outlined in the methodology. (**B**) Water consumption and (**C**) body weight were monitored daily for all experiments. (**D**) Mouse behavior and well-being were observed every day after virus inoculation and a physical condition score was calculated for each mouse. Data shown are means ± SEM and analyzed using two-way ANOVA followed by Sidak’s multiple comparison test.

In order to investigate whether tramadol treatment exerts any model-altering effects on murine CVB3-induced myocarditis, we initiated tramadol treatment via drinking water (0.1 µg/mL) one week prior to infection [22]. During the course of virus infection, the activation of the immune system and developments of virus myocarditis were examined as outlined in Figure 1A. The analysis of water consumption revealed similar drinking behavior of mice and no effect of tramadol (Figure 1B). We observed a decrease in water consumption during the first two days after CVB3 inoculation, which was reversible afterwards. Mice receiving tramadol showed a slight increase in water consumption. As observed for water consumption, the body weight of infected animals decreased markedly between day 1 and day 3 after virus inoculation in both groups of mice (Figure 1C). We observed no relevant alteration of the physical condition score during the investigational period (Figure 1D).

**Figure 2:**
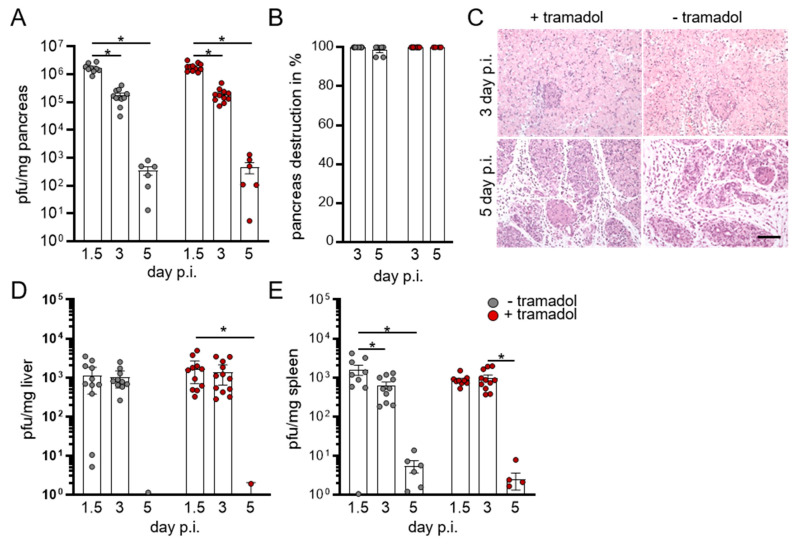
Tramadol shows no effect on virus concentration in the pancreas. (**A**) Amount of infectious virus in the pancreas on day 1.5 (*n* = 12), 3 (*n* = 12), and 5 (*n* = 6) was determined by plaque assay. (**B**) Formalin-fixed and paraffin-embedded pancreas tissue sections were stained using hematoxylin and eosin and pancreas destruction was scored. (**C**) Representative histological images of the pancreas are shown. Scale bar 50 µm. Amounts of infectious virus in (**D**) liver and (**E**) spleen on day 1.5 (*n* = 12), 3 (*n* = 12), and 5 (*n* = 6) were determined by plaque assay. Data were summarized as means ± SEM and analyzed by two-way ANOVA; * <0.05.

As shown previously, the pancreas is a primary target organ for CVB3 after intraperitoneal injection, with peak virus titer at 36 h after infection (Figure 2A). In the following days, the amount of virus subsided until no infectious particles were detected on day 8 p.i. (data not shown). Acute virus-induced pancreatitis resulted in pancreas destruction (Figure 2B,C). We detected lower titers of the infectious virus in the peripheral organs, such as liver (Figure 2D) and spleen (Figure 2E). Between days 5 and 8, CVB3 levels were no longer measurable and no virus-induced pathological alterations were observed in these organs (data not shown). Importantly, a comparison of both groups of mice revealed that the treatment with tramadol had no impact on the early phase of the virus infection in C57BL/6 mice, which is shown by the equal virus titer in the pancreas, liver, and spleen.

**Figure 3:**
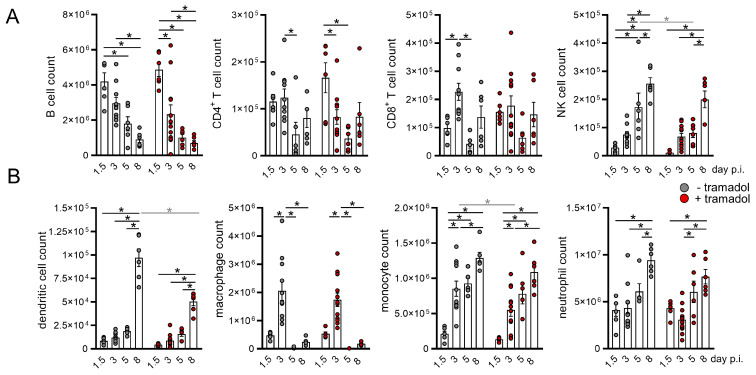
Tramadol treatment shows no perturbation of immune cells in the bone marrow. Mice were sacrificed on day 1.5, 3, 5 and 8 after Coxsackievirus B3 (CVB3) infection and cells from bone marrow were isolated from one tibia and two femurs, counted, and the number of lymphoid cells ((**A**) B cells, T cells, and natural killer cells) and myeloid cells ((**B**) dendritic cells, macrophages, monocytes, and neutrophils) were quantified by flow cytometry (d 1.5 *n* = 6, d 3 *n* = 12/11, d 5 *n* = 6, and d 8 *n* = 6). Data were summarized as means ± SEM and analyzed by two-way ANOVA; * <0.05.

**Figure 4:**
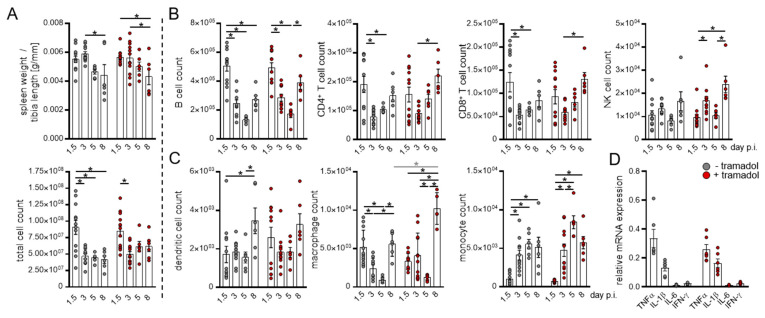
Tramadol treatment shows no perturbation on immune cells in the spleen. Mice were sacrificed on days 1.5, 3, 5, and 8 after Coxsackievirus B3 (CVB3) infection, spleens were homogenized, and single cell suspensions were subjected to immune cell quantification by flow cytometry. (**A**) Spleen weight, total number of splenocytes, and the amounts of lymphoid cells ((**B**) B cells, T cells, and natural killer cells) and myeloid cells ((**C**) dendritic cells, macrophages, monocytes, and neutrophils) were determined for each analysis day (d 1.5 *n* = 12, d 3 *n* = 11, d 5 *n* = 6, and d 8 *n* = 6). Data were summarized as means ± SEM and analyzed by two-way ANOVA. (**D**) Expression of cytokines in the spleen 3 days after infection. RNA was isolated from spleen tissues for qPCR-analysis of the indicated genes. Expression data were normalized to HPRT and calculated using the ΔCT method (*n* = 5). Data were summarized as means ± SEM and analyzed by *t* tests; * <0.05.

CVB3 efficiently activates the host immune system, starting with the type interferon (IFN) I induction via pattern recognition receptors (PRR) and the subsequent activation of innate as well as adaptive immune cells. In order to analyze the impact of tramadol on the activation of the immune system, immune cell populations and their activity state were assessed in primary (bone marrow) and secondary (spleen) lymphoid organs at different time points after infection. During CVB3 infection, a slight decrease in the number of B- and T lymphocytes at the late stage of infection (days 5 and 8) was observed, while the number of NK cells increases continuously during the course of the infection (Figure 3A). On day 5 p.i. the number of NK cells were lower in the tramadol group, but a possible tramal-induced influence on NK cells was not observed compared to other time points in the bone marrow and was completely absent in the spleen. Monocytes and their precursors increase their proliferation capacity in response to inflammatory stimuli, which is known as monocytosis. This effect was apparently prominent in the bone marrow, with a 4-fold increase in Ly6C^+^ monocytes from day 1.5 to day 3 p.i. and a further doubling by day 8 (Figure 3B). Thereby, the increase from day 1.5 to day 3 p.i. was more prominent in untreated mice. Moreover, an increasing number of dendritic cells (DC) and neutrophils was measured during the course of infection, which indicates active myelopoiesis as a reaction to virus-induced inflammatory processes. For dendritic cells, tramadol seemed to interfere with this adaptation 8 days after infection, since in the tramadol group CD11c^+^ bone marrow cell counts were lower in comparison to untreated mice (Figure 3B). Nevertheless, this observation was not reflected in any effects, e.g., on DC counts or in the spleen (Figure 4C), and occurred after the activation peak of DCs in both the bone marrow and the spleen (Table 1, Table 2, Table 3 and Table 4).

Tramadol treatment had no continuous effect on immune cell populations in these experiments. As previously described [25], CVB3 infection resulted in spleen atrophy with decreased lower total amounts of splenocytes (Figure 4A) in both groups. A similar temporary effect was also visible for lymphocytes (Figure 4B). Compared to the mock treated mice, animals that received tramadol showed a slight but not significant increase in the total amount of splenocytes and lymphocytes, suggesting a possible immune-modulating effect of tramadol. However, this was not observed for the bone marrow where the trend was observed exemplify the opposite (Figure 3A,B). More prominent than the quantitative differences of lymphoid cells in the spleen and bone marrow are the changes in their activation state (Table 1 and Table 3). High amounts of CD44^+^ and CD69^+^ T-lymphocytes are present in both lymphatic organs in the early phase of infection. In particular, CD69^+^ CD4^+^ T-cells are markedly upregulated on day 3 p.i. in both the spleen and bone marrow, with a 3-fold to 5-fold increase compared to day 1.5. Similar results were observed for myeloid cells. While the amounts of macrophages and dendritic cells in the spleen (Figure 4C) and bone marrow (Figure 3C) display no collective trend during infection, the presence of mature populations expressing MHCII and CD86 are significantly increased during the early phase of infection (day 1.5 and d 3) p.i. in both organs (Table 2 and Table 4). On day 8 p.i. an increase in macrophages in tramadol treated mice was measured; this is an observation that was not confirmed for other time points or myeloid cell populations in the spleen. In this stage the inflammations and immune cell activations are subsided (Table 4, Figure 4D). 

The increasing presence of activated immune cells during the early phase of infection, especially on day 3 p.i., further resulted in an abundant IL-1β and TNF-α expression in the spleen (Figure 4D), similar to that measured in the heart. In conclusion, analysis of both lymphoid organs showed that differences in immune cell populations between both treatment groups, quantitative as well as activation-related, were not consistent.

**Figure 5:**
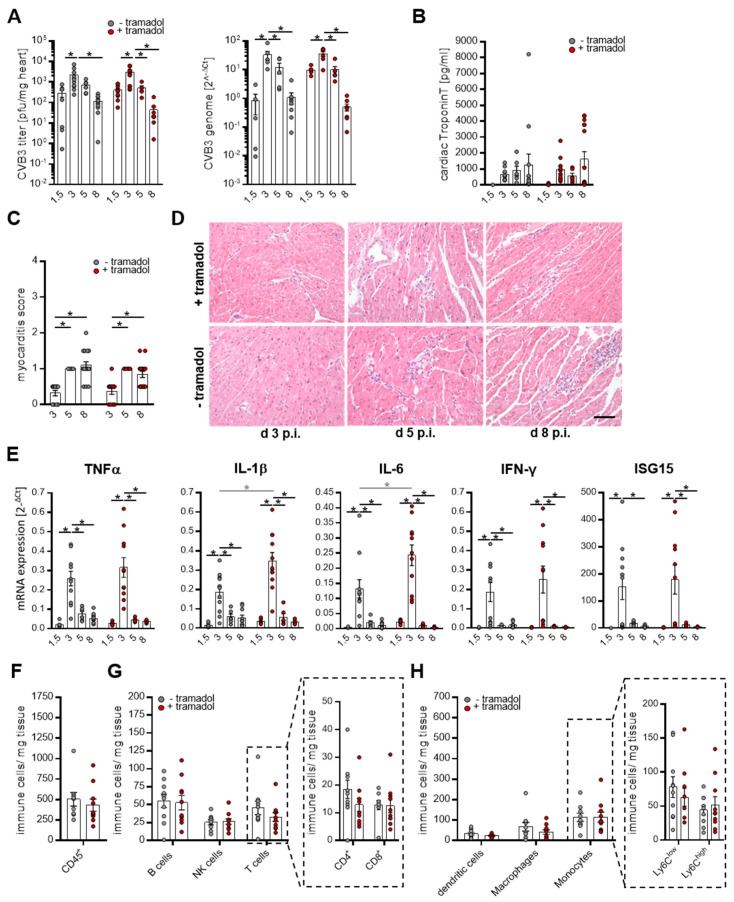
Tramadol has no impact on Coxsackievirus B3 (CVB3)-induced myocarditis. (**A**) Quantification of viral load in the heart. Mice were sacrificed on days 1.5, 3, 5, and 8 after CVB3 infection and the viral load was determined by the quantification of infectious viral particles by plaque assay (left graph) and viral genomes using quantitative PCR (right graph). (**B**) Blood samples were taken on the different analysis days and cardiac troponin T was measured (d 1.5 *n* = 6, d 3 *n* = 11, d 5 *n* = 6, and d 8 *n* = 16). (**C**,**D**) Inflammation and necrosis in the heart. Formalin-fixed and paraffin-embedded heart tissue sections were stained with hematoxylin/eosin and myocarditis severity was semi-quantified using a scoring system ranging from 0 to 4 (d 3 *n* = 12/11, d 5 *n* = 6, and d 8 *n* = 16). (**D**) Representative images of the heart sections for each group and time point are shown. Scale bar 50 µm. (**E**) mRNA expression of the indicated genes in infected hearts. Expression data were normalized to HPRT and calculated using ΔCT method (d 1.5 *n* = 6, d 3 *n* = 11, d 5 *n* = 6, and d 8 *n* = 10). Data were summarized as means ± SEM and analyzed by two-way ANOVA. (**F**–**H**) Quantification of immune cell infiltration in the heart 8 days after infection. Single cell suspensions from heart tissue were generated and (**F**) lymphoid and (**G**) myeloid cells were quantified by flow cytometry. Values are shown as mean ± SEM (*n* = 10). Data were analyzed by *t* tests; * <0.05.

The most important part of this study is the examination of possible model-altering effects of tramadol with regard to the manifestation of CVB3-induced myocarditis. Therefore, cardiac virus concentration, virus-induced cardiac tissue damage, and the virus-triggered immune response were analyzed. In both mouse groups, plaque assay (Figure 5A left) and quantification of viral genomes (Figure 5A right) demonstrated similar virus levels in the myocardium on day 1.5. They further revealed enhanced virus titer after 3 days and persisting virus presence until day 8 in both groups. Cardiac damage was examined by the histological analysis of heart tissue (Figure 5C,D). Minor histological alterations were observed on day 3 p.i., which increases in severity until day 8 and are characterized by the inflammation of mononuclear cell infiltrates and tissue damage. Differences in virus-induced myocardial tissue injury between the tramadol-treated and un-treated mice were not observed. 

We profiled the cardiac mRNA expression for the pro-inflammatory cytokines IL-6, IL-1β and TNF-α, the Interferon stimulated gene 15 (ISG15) which is an effector of the type I interferon response with antiviral properties, and IFN-γ and found peak levels on day 3 p.i. that subsided by day 8 (Figure 4E). Slightly increased cytokine expression levels in tramadol-treated mice were observed on day 3, hinting at a minor immune modulating activity of tramadol, but possible effects disappear as the infection progresses. In response to pathogens, the cytokines play an important role in recruiting and activating immune cells in the infected tissue. Since immune cell infiltration is a hallmark of the myocarditis model, quantitative analyses of lymphoid (Figure 5G) and myeloid (Figure 5H) cells in cardiac tissue were performed by flow cytometry on day 8 after virus infection. The amount of CD45^+^ immune cells in the heart was similar in both treatment groups (Figure 5F). Furthermore, the abundances of myeloid and lymphoid cells were equivalent in both groups and thus an influence of tramadol treatment on cardiac immune cell infiltration was not observed. Similar results were found for cardiac functional parameters measured by echocardiography on days 0 and 8 p.i. (Table 5), where no differences between the groups of mice were documented. Taken together, these results demonstrate that tramadol possesses no model-altering impact on the CVB3-induced acute myocarditis in C57BL/6 mice.

## 4. Discussion

Animal studies have contributed much to our current knowledge of etiology, progression, and molecular pathogenesis of myocarditis. They further increased our understanding of the role of the immune system in cardiac inflammatory disease and represents an essential tool for the development and testing of causative as well as symptomatic therapies [26]. In recent years, there has been increasing consideration of the use of analgesia in animal infection models with a severe disease burden, such as the CVB3 infection model. Although the increasing emphasis on animal welfare is legitimate, the integrity of the animal model has to be ensured and therefore strategies to improve animal welfare require appropriate in vivo testing. Almost all analgesics, such as nonsteroidal anti-inflammatory drugs and opioids, are thought to interact with the immune system and thus affect one of the main model-defining hallmarks needed for the proper manifestation of CVB3-triggered myocarditis [16,27]. Little has been conducted to study the impact of opioids on the murine immune system and no data exist for the impact of analgesics on CVB3-myocarditis. Therefore, we analyzed the impact of tramadol in CVB3-infected C57BL/6 mice, a mouse strain that is often used to study acute viral myocarditis. We demonstrated that tramadol, which is a synthetic opiate used for analgesia in humans and animals, exerts no immune modulating impact that affects the murine CVB3-induced myocarditis model.

In our study, tramadol was given seven days prior to CVB3 infection to avoid previously described immune modifications that were detected directly after tramadol administration [22]. We propose such a precondition phase with tramadol treatment prior to infection as a general concept for the CVB3 myocarditis model. The infection with CVB3 Nancy in C57BL/6 mice, as presented here, had no relevant effect on the physical condition score and mice developed a mild disease. This ultimately resulted in the fact that the analgesic effect by tramadol could not be scaled directly in our model. Nevertheless, we used the dosage of 0.1 mg/mL tramadol, for which a profound analgesic effect was proven earlier in mice after applying an osteotomy model. In this model, tramadol did not induce well-known opioid-related side effects such as reduced drinking behavior or decreased body weight [23]. In accordance with these data, we did not observe a drop in the water intake of the tramadol-treated mice compared to mice receiving only water. It is known that opioid analgesics may affect cardiac function with decreased blood pressure, heart rate, and cardiac output. This was not observed for tramadol in humans [28] and our analysis of cardiac function in both mice groups also revealed no effect of tramadol. 

As a mode of their action, opioids interact with the immune system [27]. On the one hand, the immune system is important during CVB3 myocarditis for viral elimination by directly recognizing and killing infected cells [13] and possible immunosuppressive effects by opioids could encourage infection by viruses. A worsened disease outcome resulting from increased virus replication could be the consequence. Quantification of infectious virus in the pancreas, heart, liver, and spleen on different days after infection revealed no effect of tramadol on virus load or on virus clearance. As mentioned earlier, the immune system in the virus-induced myocarditis is not only necessary for effective virus clearance, but further induces model-defining cardiac damage by immune cell infiltration and other inflammatory processes. In the few studies which used tramadol as an analgesic in rodents, both anti-inflammatory [29] as well as pro-inflammatory [22,30] effects have been described. Thereby Sacerdote et al. mentioned an increased proliferation of splenocytes after acute tramadol application, but in a chronic study in which tramadol was given over 2 weeks this effect disappeared. In accordance with this observation, no differences in the total amount of splenocytes were measured between the tramadol-treated mice and the control group. Furthermore, as a reaction to the CVB3 infection, a similar decrease in total splenocytes was observed in both treatment groups, which is an effect that has previously been described [25]. A detailed analysis of the different lymphoid and myeloid immune cell populations in the spleen and bone marrow did not reveal any significant differences between the two groups. Moreover, the increasing maturation of antigen presenting cells and subsequent activations of T cells during early virus infection were found to be unchanged in tramadol-treated and untreated mice. Furthermore, infection-related cytokine expression in the spleen was not altered by tramadol, as has been shown for other opioids [31]. In experiments in rats, Bianchi M. et al. found that tramadol led to less edema after yeast injection and less Carrageenin-induced inflammatory exudates [29]. In both models, tramadol was given a few hours before the experimental intervention. In our study no signs of anti-inflammatory effects were found, however, indicating any immune-modulating impact of tramadol is transient and no longer plays a role with this chronic treatment regime. Similar to the unaffected virus-induced immune activation in the spleen and bone marrow, no differences in cardiac immune cell infiltration were observed on day 8 after infection. Moreover, cardiac damage and myocarditis score, which are the most important parameters for the evaluation of myocarditis severity, remain unaffected by tramadol.

In conclusion, our study analyzed for the first time the application of tramadol in the murine CVB3-myocarditis model, with a particular focus on the activation of the immune system and cardiac pathogenesis. No alterations of immune cell populations and their activation in the spleen, bone marrow, and heart or modified cytokine expression, virus presence, and, most importantly, heart pathology were observed when tramadol was administered chronically seven days before infection. This shows that tramadol can be considered as an analgesic for CVB3 infection in mice and this provides the opportunity to increase animal welfare without provoking disruptive effects on the key aspects of the CVB3 myocarditis model.

## Figures and Tables

**Table 1:** CD44/CD69 expression on lymphoid cells in bone marrow.

	CD4^+^ T Cells				CD8^+^ T Cells			
	CD44^+^		CD69^+^		CD44^+^		CD69^+^	
Tramadol	+	−	+	−	+	−	+	−
d 1.5 p.i.	92.8 ± 2 *	95.7 ± 1 *	11.5 ± 2 ^$^	6.7 ± 3 ^$^	69.9 ± 3 *	69.7 ± 4 *	51.9 ± 3	25.1 ± 10 ^#^
d 3 p.i.	85.9 ± 2 *	91.1 ± 1 *	43 ± 3	34.8 ± 2	73.7 ± 3 *	76.7 ± 2 *	48.6 ± 5	45.3 ± 6
d 5 p.i.	87.1 ± 3 *	78.9 ± 4 *	16.3 ± 2 ^$^	11.5 ± 2 ^$^	71.9 ± 3 *	61.3 ± 2 *	22 ± 1.4 ^$,§^	15.7 ± 1 ^$^
d 8 p.i.	45.6 ± 9	49.3 ± 12	15.9 ± 5 ^$^	15.5 ± 7 ^$^	40.3 ± 7	40.2 ± 10	14.4 ± 3 ^$,§^	10.2 ± 2 ^$^

Tramadol-treated and untreated C57BL/6 mice were infected with Coxsackievirus B3 (CVB3). On day 1.5, 3, 5, and 8 after infection, single cell suspensions from the bone marrow were analyzed by flow cytometry to quantify the expression of CD44 and CD69 in lymphoid populations. Data shown are mean ± SEM and were analyzed using two-way ANOVA. * indicates significant differences compared to day 8 within the same treatment group. ^$^ indicates significant differences compared to day 3 within the same treatment group. ^§^ indicates significant differences compared to day 1.5 within the same treatment group. ^#^ indicates significant differences between the + tramadol and − tramadol group.

**Table 2:** MHC class II/CD86 expression on myeloid cells in bone marrow.

	Dendritic Cells				Macrophages			
	MHC II^+^		CD86^+^		MHC II^+^		CD86^+^	
Tramadol	+	−	+	−	+	−	+	−
d 1.5 p.i.	21.9 ± 2 ^$^	31.9 ± 5	31.9 ± 2	22.3 ± 3	3.9 ± 0.4 ^$^	3 ± 1 ^$^	47.9 ± 2	26 ± 6
d 3 p.i.	37.4 ± 4	40.5 ± 3	24.7 ± 3	23 ± 3	23.6 ± 5	27.3 ± 5	38.5 ± 4	38.9 ± 4
d 5 p.i.	21.2 ± 3 ^$^	18.4 ± 2 ^$^	3 ± 0.6 ^§,$^	4.5 ± 0.8 ^§,$^	5.1 ± 0.3 ^$^	5.8 ± 1 ^$^	2.2 ± 0.3 ^§,$^	3.6 ± 0.9 ^§,$^
d 8 p.i.	18.1 ± 2 ^$^	17.6 ± 2 ^$^	0.2 ± 0.02 ^§,$^	0.1 ± 0.04 ^§,$^	1.8 ± 0.3 ^$^	1.8 ± 0.2 ^$^	0.2 ± 0.05 ^§,$^	0.1 ± 0.03 ^§,$^

Tramadol-treated and untreated C57BL/6 mice were infected with Coxsackievirus B3 (CVB3). On day 1.5, 3, 5, and 8 after infection, single cell suspensions from bone marrow were analyzed by flow cytometry to quantify the expression of MHC class II molecules and CD86 in myeloid populations. Data shown are mean ± SEM and were analyzed using two-way ANOVA. ^$^ indicates significant differences compared to day 3 within the same treatment group. ^§^ indicates significant differences compared to day 1.5 within the same treatment group.

**Table 3:** CD44/CD69 expression on lymphoid cells in spleen.

	CD4^+^ T Cells				CD8^+^ T Cells			
	CD44^+^		CD69^+^		CD44^+^		CD69^+^	
Tramadol	+	−	+	−	+	−	+	−
d 1.5 p.i.	43.3 ± 2	48.2 ± 2	4.3 ± 0.4	6 ± 1	55.2 ± 2	57.1 ± 3	7.6 ± 0.8	15.5 ± 4
d 3 p.i.	39.2 ± 3	37.6 ± 3 ^§^	20.8 ± 2 ^§^	19.1 ± 2 ^§^	49.9 ± 2	49.9 ± 1 ^§^	18.5 ± 5	18.2 ± 5
d 5 p.i.	19.8 ± 1 ^§,$^	17.9 ± 0.8 ^§,$^	6.4 ± 0.3 ^$^	5.7 ± 0.6 ^$^	28.6 ± 1 ^§,$^	29.5 ± 1 ^§,$^	3.7 ± 0.3	3.6 ± 0.2
d 8 p.i.	23.5 ± 1 ^§,$^	23.6 ± 1 ^§,$^	12.3 ± 1 ^§,$^	9.2 ± 0.7 ^§,$^	30.2 ± 1 ^§,$^	30.2 ± 1 ^§,$^	8 ± 1	5.8 ± 0.4

Tramadol-treated and untreated C57BL/6 mice were infected with Coxsackievirus B3 (CVB3). On day 1.5, 3, 5, and 8 after infection, single cell suspensions from spleen tissue were analyzed by flow cytometry to quantify the expression of CD44 and CD69 in lymphoid populations. Data shown are mean ± SEM and were analyzed using two-way ANOVA. ^$^ indicates significant differences compared to day 3 within the same treatment group. ^§^ indicates significant differences compared to day 1.5 within the same treatment group.

**Table 4:** MHC class II/CD86 expression on myeloid cells in spleen.

	Dendritic Cells				Macrophages			
	MHC II^+^		CD86^+^		MHC II^+^		CD86^+^	
Tramadol	+	−	+	−	+	−	+	−
d 1.5 p.i.	23.4 ± 2 *	32.6 ± 3 *	23.8 ± 5 *	25.9 ± 2 *	10.4 ± 2 ^$^	22.6 ± 3	16.5 ± 4 ^$^	22.8 ± 2 ^$^
d 3 p.i.	34.1 ± 6 *	30.4 ± 4 *	31.4 ± 6 *	29.8 ± 5 *	32.5 ± 6	34.1 ± 6	44.5 ± 9	44.3 ± 9
d 5 p.i.	29 ± 3 *	21.3 ± 2 *	17.3 ± 0.8	13.7 ± 1	18.1 ± 1	14.5 ± 1.5 ^$^	25.6 ± 1	23.4 ± 2
d 8 p.i.	2.9 ± 0.4	2.3 ± 0.2	1.3 ± 0.2	1.3 ± 0.2	8.2 ± 1 ^$^	9.9 ± 1 ^$^	0.5 ± 0.1^$^	0.6 ± 0.2 ^$^

Tramadol-treated and untreated C57BL/6 mice were infected with Coxsackievirus B3 (CVB3). On day 1.5, 3, 5, and 8 after infection, single cell suspensions from spleen tissue were analyzed by flow cytometry to quantify the expression of MHC class II molecules and CD86 in myeloid populations. Data shown are mean ± SEM and were analyzed using two-way ANOVA. * indicates significant differences compared to day 8 within the same treatment group. ^$^ indicates significant differences compared to day 3 within the same treatment group.

**Table 5:** Analysis of cardiac performance.

	− Tramadol		+ Tramadol	
	Baseline	Day 8	Baseline	Day 8
Heart rate (bpm)	480 ± 9.2	422 ± 20 *	457 ± 10.7	410 ± 22
Trace EF (%)	53.7 ± 1.2	52.7 ± 2.5	55.1 ± 1.3	51.1 ± 2.4
Cardiac output (mL/min)	11.2 ± 0.4	10.0 ± 1	9.9 ± 0.8	10 ± 0.8
Stroke volume (µL)	23.2 ± 0.8	22.8 ± 1.5	21.4 ± 1.6	24.3 ± 1.4
Vol d (µL)	43.4 ± 1.4	43.9 ± 1.9	39.5 ± 2.4	45.5 ± 1.7
Vol s (µL)	20.2 ± 0.9	21 ± 1.4	18.1 ± 1	21.2 ± 1.3
LVID-d (mm)	3.7 ± 0.04	3.7 ± 0.07	3.7 ± 0.06	3.8 ± 0.05
LVID-s (mm)	2.7 ± 0.06	2.8 ± 0.08	2.7 ± 0.06	2.8 ± 0.09

Tramadol-treated and untreated C57BL/6 mice were infected with CVB3. Echocardiography was performed at baseline (day 0) and after 8 days, using a Vevo 3100. Data shown are mean values ± SEM and were analyzed using two-way ANOVA. * indicates a significant change (*p* < 0.05) between day 0 and day 8 p.i.; *n* = 16. EF = ejection fraction; bpm = beats per minute; Vol d/s = end-diastolic/systolic left ventricular volume; LVID-d/s = left ventricular diameter at diastole/systole.

## Supplementary Materials

The following are available online at https://www.mdpi.com/article/10.3390/v13071222/s1, Figure S1: Gating strategy of lymphoid cells, Figure S2: Gating strategy of myeloid cells, Figure S3: Gating strategy of heart cells.
