# Supplementary material for: Exploration of Analgesia with Tramadol in the Coxsackievirus B3 Myocarditis Mouse Model
Source: Viruses. 2021 Jun 24;13(7):1222. doi: 10.3390/v13071222 (PMC8310306; doi:10.3390/v13071222)
Supplement: Supplementary file 1 [file viruses-13-01222-s001.zip › viruses-1241335-supplementary.pdf]

Supplemental figure S1

lymphoid cells

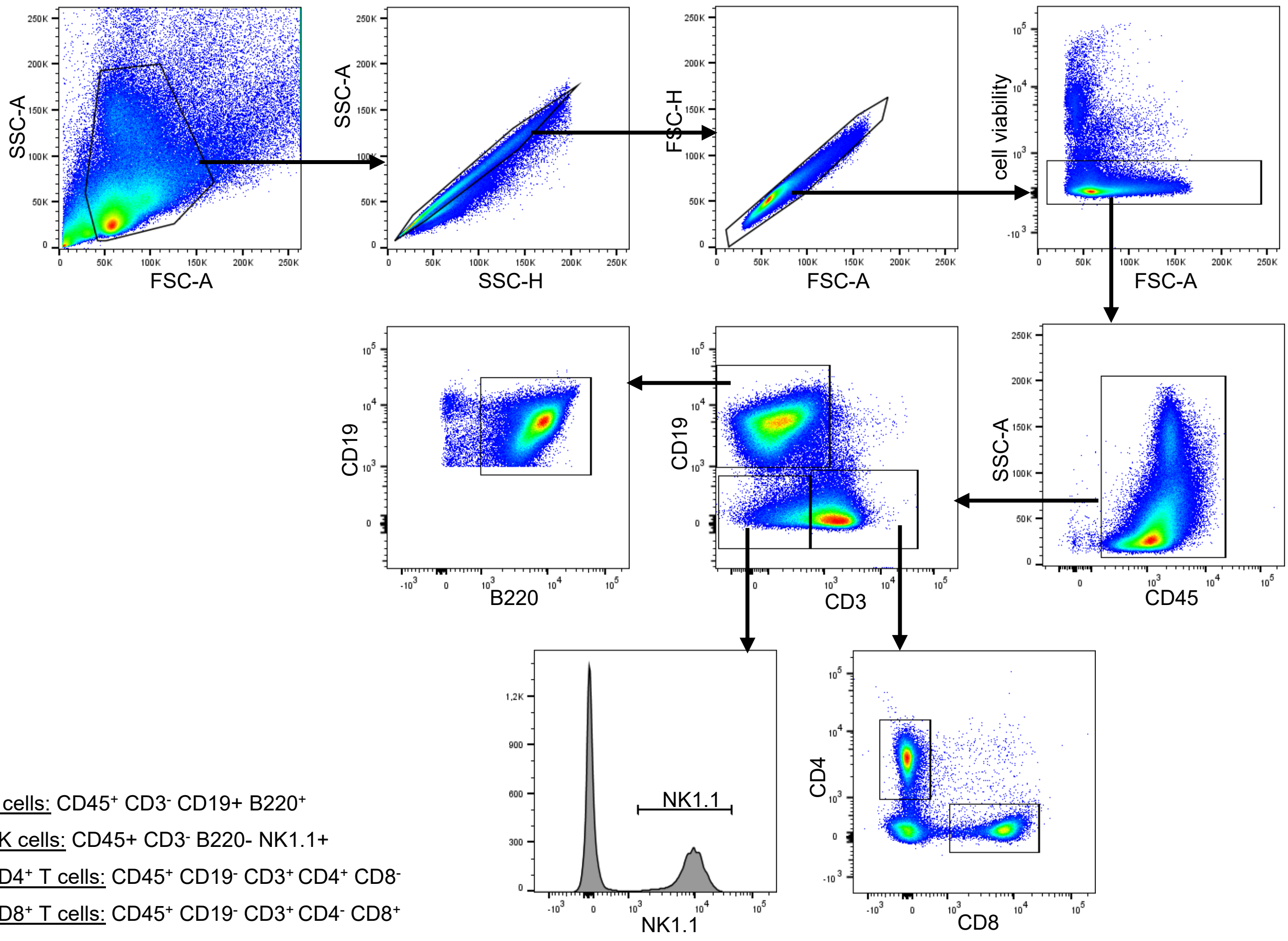

B cells: CD45$^+$ CD3$^-$ CD19+ B220$^+$

NK cells: CD45+ CD3$^-$ B220- NK1.1+

CD4$^+$ T cells: CD45$^+$ CD19$^-$ CD3$^+$ CD4$^+$ CD8$^-$

CD8$^+$ T cells: CD45$^+$ CD19$^-$ CD3$^+$ CD4$^-$ CD8$^+$

Supplemental figure S2

myeloid cells

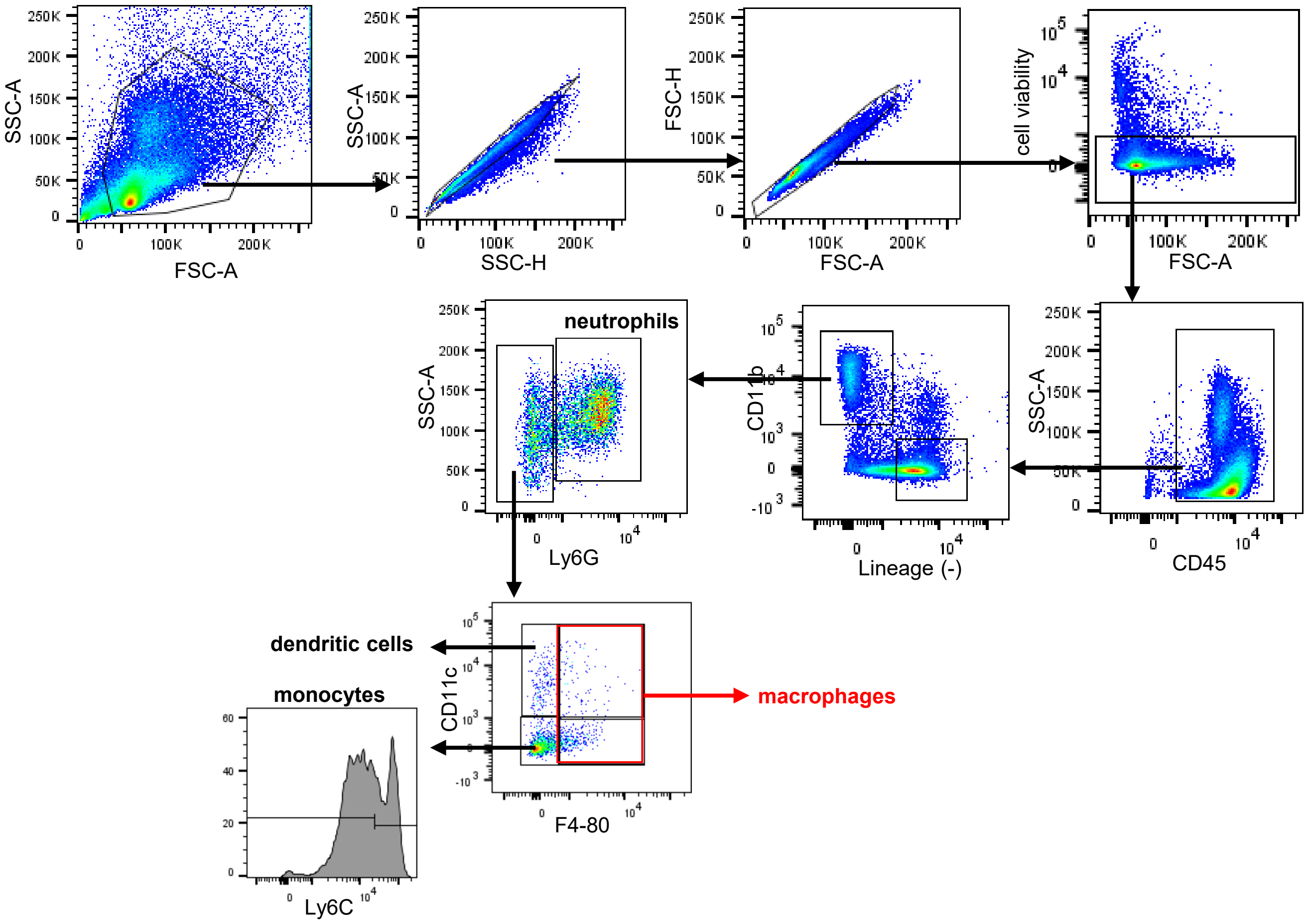

neutrophils: $CD45^+$ $Lineage^–$ ($CD3^-$ $B220^-$ $Ter119^-$ $CD49b^-$) Ly6G+

dendritic cells (DCs): $CD45^+$ $Lineage^–$ ($CD3^-$ $B220^-$ $Ter119^-$ $CD49b^-$) $Ly6G^-$ $CD11c^+$ $F4\text{-}80^-$

macrophages: $CD45^+$ $Lineage^–$ ($CD3^-$ $B220^-$ $Ter119^-$ $CD49b^-$) $Ly6G^-$ $CD11c^{+/-}$ $F4\text{-}80^+$

Ly6C+ monocytes: $CD45^+$ $Lineage^–$ ($CD3^-$ $B220^-$ $Ter119^-$ $CD49b^-$) $Ly6G^-$ $CD11c^-$ $F4\text{-}80^-$ $Ly6C^+$

Supplemental figure S3

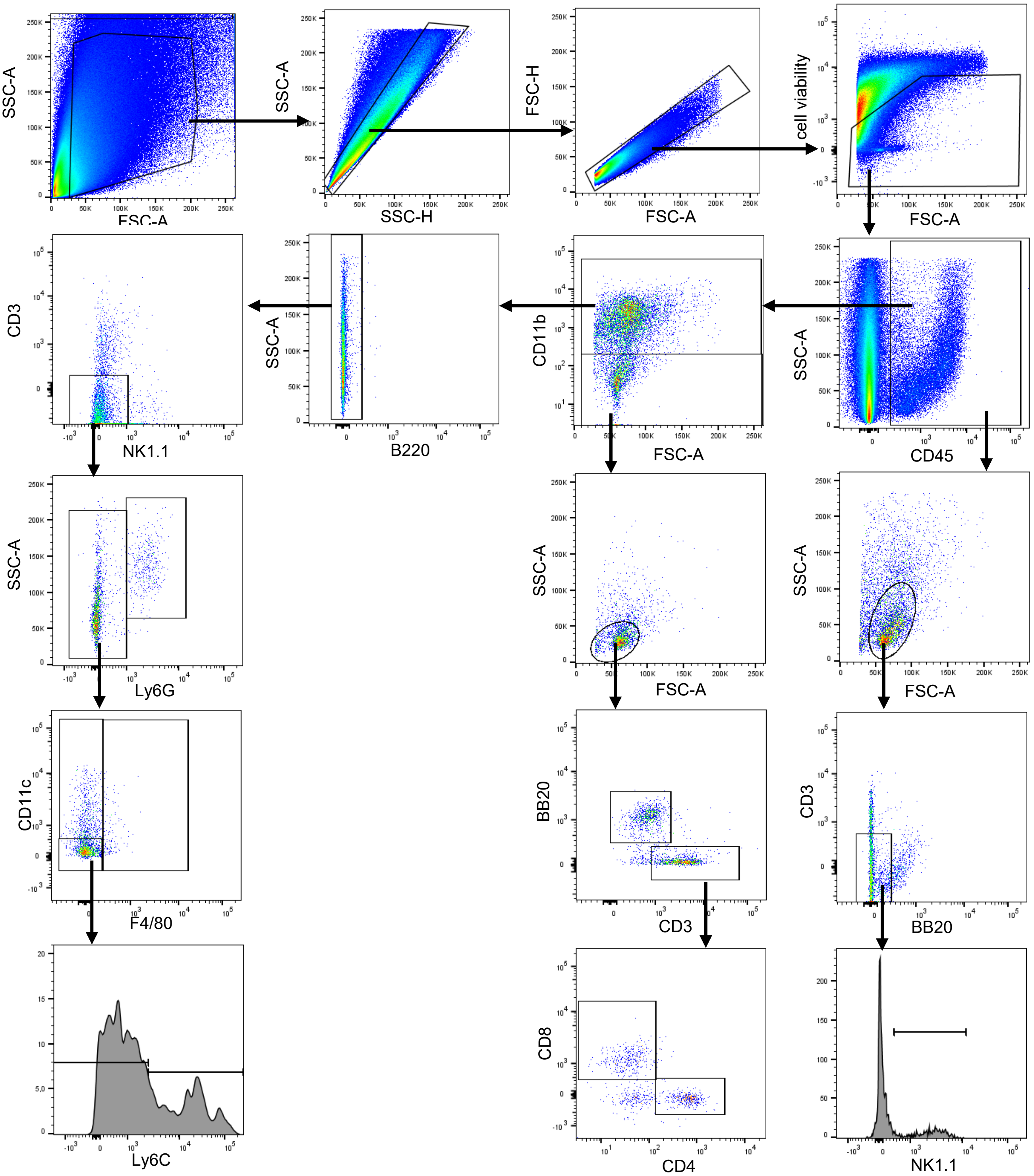

Macrophages: CD45⁺ CD11b+ B220⁻ CD3⁻ NK1.1⁻ Ly6G⁻ CD11c$^{+/-}$ F4/80⁺

Dendritic cells: CD45⁺ CD11b+ B220⁻ CD3⁻ NK1.1⁻ Ly6G⁻ CD11c⁺ F4/80⁻

Ly6^{hi} monocytes: CD45⁺ CD11b+ B220⁻ CD3⁻ NK1.1⁻ Ly6G⁻ CD11c⁻ F4/80⁻ Ly6C^{hi}

Ly6^{low} monocytes: CD45⁺ CD11b+ B220⁻ CD3⁻ NK1.1⁻ Ly6G⁻ CD11c⁻ F4/80⁻ Ly6C^{low}

B cells: CD45⁺ CD11b⁻ B220⁺ CD3⁻ / NK cells: CD45+ CD3- B220- NK1.1+

CD4⁺ T cells: CD45⁺ CD11b⁻ B220⁻ CD3⁺ CD4⁺ CD8⁻ / CD8⁺ T cells: CD45⁺ CD11b⁻ B220⁻ CD3⁺ CD4⁻ CD8⁺
